# SDAR: a practical tool for graphical analysis of two-dimensional data

**DOI:** 10.1186/1471-2105-13-201

**Published:** 2012-08-14

**Authors:** Saroja Weeratunga, Nien-Jen Hu, Anne Simon, Andreas Hofmann

**Affiliations:** 1 Structural Chemistry Program, Eskitis Institute for Cell and Molecular Therapies, Griffith University, Brisbane, Qld, 4111, Australia; 2 Division of Molecular Biosciences, Imperial College London, London, SW7 2AZ, UK; 3 Universite Lyon 1, CNRS ICBMS 5246, Institut de Chimie et Biochimie Moleculaires et Supramolaires, Laboratoire Genie Enzymatique, Membrane Biomimetique et Assemblages Supramoleculaires, F-69622, Villeurbanne, France; 4 Faculty of Veterinary Science, The University of Melbourne, Parkville, VIC, 3010, Australia; 5 Queensland Tropical Health Alliance, Townsville, Qld, 4811, Australia

## Abstract

**Background:**

Two-dimensional data needs to be processed and analysed in almost any experimental laboratory. Some tasks in this context may be performed with generic software such as spreadsheet programs which are available ubiquitously, others may require more specialised software that requires paid licences. Additionally, more complex software packages typically require more time by the individual user to understand and operate. Practical and convenient graphical data analysis software in Java with a user-friendly interface are rare.

**Results:**

We have developed SDAR, a Java application to analyse two-dimensional data with an intuitive graphical user interface. A smart ASCII parser allows import of data into SDAR without particular format requirements. The centre piece of SDAR is the Java class *GraphPanel* which provides methods for generic tasks of data visualisation. Data can be manipulated and analysed with respect to the most common operations experienced in an experimental biochemical laboratory. Images of the data plots can be generated in SVG-, TIFF- or PNG-format. Data exported by SDAR is annotated with commands compatible with the Grace software.

**Conclusion:**

Since SDAR is implemented in Java, it is truly cross-platform compatible. The software is easy to install, and very convenient to use judging by experience in our own laboratories. It is freely available to academic users at http://www.structuralchemistry.org/pcsb/. To download SDAR, users will be asked for their name, institution and email address. A manual, as well as the source code of the *GraphPanel* class can also be downloaded from this site.

## Background

Data analysis and processing are tasks met in almost any experimental laboratory. Widely used software for such tasks include ubiquitous generic spreadsheet programs such as MS Excel, as well as sophisticated commercial software packages such as SigmaPlot, Origin, IGOR, etc. In the last ten years, free and open source software has also been developed, mainly based on C++ and Python. This includes, but is not limited to, software such as Fityk [1], peak-o-mat ( http://lorentz.sourceforge.net/), HippoDraw ( http://www.slac.stanford.edu/grp/ek/hippodraw/index.html), Veusz ( http://home.gna.org/veusz/), ParaView ( http://www.paraview.org/), gnuplot ( http://www.gnuplot.info), R ( http://www.R-project.org) and others. One of the most established software in this respect is Grace ( http://plasma-gate.weizmann.ac.il/Grace/), a descendant of the ACE/gr 2D plotting tool originally developed for Unix.

Our lab has been developing practical Java applications focused on structural biology tasks since 2002 [2-5]. Numerical methods well established in the classical scientific programming languages such as Fortran and C have increasingly been developed and implemented in Java. For instance, JAMA provides classes for constructing and manipulating real matrices and their decompositions ( http://math.nist.gov/javanumerics/jama/), and many algorithms have been made available by developers in Java, including the very extensive library of scientific and numerical classes by Flanagan ( http://www.ee.ucl.ac.uk/~mflanaga/java/). Despite the availability of numerical methods implementations, there is a surprising lack of interface-oriented Java software for data analysis and processing. According to a list of numerical analysis software in Wikipedia    ( http://en.wikipedia.org/wiki/List_of_numerical_analysis_software; update as of 16 Feb 2012), there is only one Java/Jython program listed in this context, namely jHepWork ( http://jwork.org/jhepwork/).

Based on Java classes developed within our Program Collection for Structural Biology and Biophysical Chemistry [2], we set out to design a simple-to-use and portable Java application for Serial Data Analysis and Regression (SDAR), which enables graphical visualisation, transformation and fitting of two-dimensional data. The emphasis of this application has been intuitive usability and quick access to a variety of laboratory-derived raw data. Concomitantly, a class handling the 2D plotting (*GraphPanel*) has also been programmed.

## Implementation

**Figure 1:**
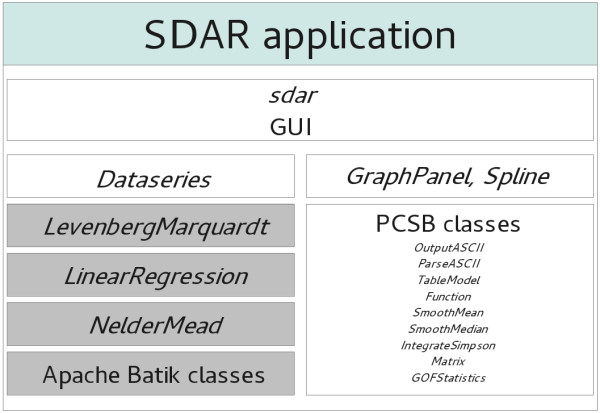
**Schematic composition of the SDAR Java application.** The four PCSB components (white) of the program are the main class providing the GUI (*sdar*), a class to describe the *Dataseries* objects, the JPanel class *GraphPanel* that provides plotting functionality, and supporting PCSB classes. SDAR also uses classes for Levenberg-Marquardt minimisation (implemented by JP Lewis), linear regression and Nelder-Mead simplex non-linear regression (implemented by M Flanagan), as well as the Apache Batik SVG toolkit (grey).

SDAR is a Java application that builds on and extends fundamental Java classes developed within the Program Collection for Structural Biology and Biophysical Chemistry (PCSB) [2]. The four PCSB components of the program are the main class providing the GUI, a class to describe the *Dataseries* objects, a JPanel class *GraphPanel* that provides plotting functionality, and general classes from our PCSB library (Figure 1). The SDAR source code is available upon request.

For curve fitting, Levenberg-Marquardt minimisation (implemented in Java by JP Lewis; http://scribblethink.org/index.html) and regression methods provided by the Flanagan library ( http://www.ee.ucl.ac.uk/~mflanaga/java/Regression.html) have been implemented, depending on the type of equation. These methods are implemented in SDAR as classes *LevenbergMarquardt*, *LinearRegression* and *NelderMead*. In order to generate scalable vector graphics (SVG) images, SDAR uses the Apache Batik SVG toolkit  ( http://xmlgraphics.apache.org/batik/index.html) which also allows for generation of image files in PNG- and TIFF-format.

## Results

SDAR uses tabbed panels to enable viewing of datasets. The main panel tabbed *Graph* shows graphical x-y-plots of the current datasets. For each dataset, a new tabbed panel is added with the name of the set showing as label in the tab. These latter panels show the spreadsheet format of the dataset, comprising of the x-y-data in the first columns, as well as any data derived from analysis in SDAR in the following columns. At the bottom of these panels, two functions are provided: *Close* will delete this dataset from the current session, *Save* writes the current dataset to an ASCII file compatible with the format of the program Grace; data derived from analysis in SDAR will be saved as remarks (indicated by #) at the top of the file. In the table view, the user can change data entries in the first two columns. With *Update*, the amended data get plotted in the *Graph* panel (note that in order to save the amended data, the *Save* option still needs to be executed).

A movable tool bar allows quick access to frequently used graphics functions.

The current session can be saved in a binary file using the *File*-*Save*, and retrieved with the *File*-*Load* function.

On the *Graph* panel, vertical and horizontal line cursors can be activated and positioned using either a click with the left mouse button, or the arrow keys UP, DOWN, LEFT and RIGHT. The x- and y-values of the active dataset at the current cursor position are displayed in the status bar. Transformations can be applied simultaneously to any selection of datasets.

The transformation features implemented in the current version of SDAR include:

• Translation of data series in x- or y-direction

• Scaling of x- or y-values

• Change data pitch (omission of data points)

• Addition of a linear function to data series

• Smoothing of data series, using the mean or median of sliding windows

The current analysis features include:

• Automatic and manual determination of maxima and minima

• Integration

Curve fitting of data series is currently possible with the following functions:

• Linear (manual, *Linear Regression*, *LevenbergMarquardt*)

• Sigmoid (manual, *NelderMead*, *LevenbergMarquardt*)

• Hill (manual, *NelderMead*, *LevenbergMarquardt*)

• Hill with background (manual, *NelderMead*, *LevenbergMarquardt*)

• Dose–response/logistic EC50 (manual, *NelderMead*, *LevenbergMarquardt*)

• Gaussian (manual, graphically, *NelderMead*, *LevenbergMarquardt*)

• Exponential (manual, *NelderMead*, *LevenbergMarquardt*)

**Figure 2:**
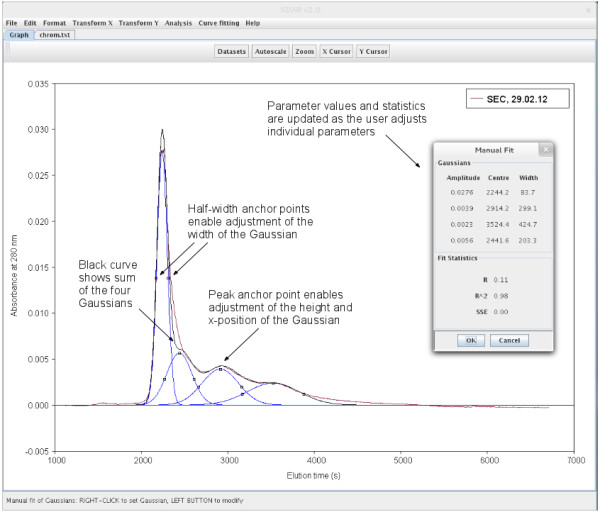
**Screen shot of a graphical Gaussian curve fitting example in SDAR.** Shown is the fitting of elution peaks obtained in a size exclusion chromatogram with four Gaussian functions. The user can place a Gaussian function with a mouse right-click at a particular x-position in the graph. Using the peak and half-width anchor points which can be dragged by a mouse left-click, the Gaussians can be adjusted to fit the experimental peaks. The parameter values as well as the goodness of fit of the resulting sum curve (black) are updated in real time in the inset window.

**Figure 3:**
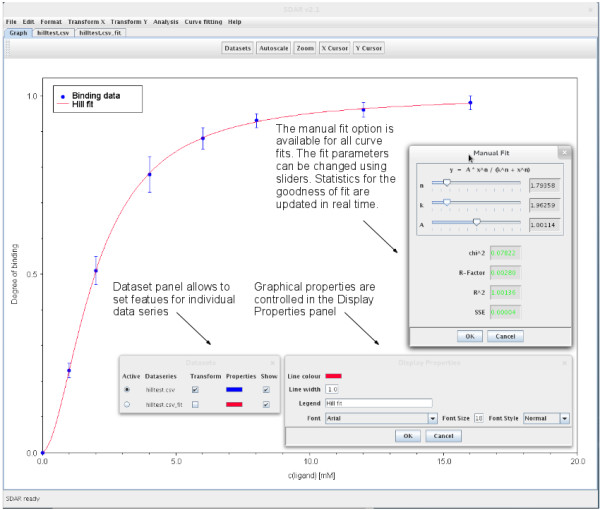
**Screen shot of a manual curve fitting example for the Hill equation in SDAR.** The experimental data are shown as discrete plot with error bars in blue. The red curve is a fit of the Hill equation to the experimental data using the non-linear Simplex algorithm. The user can adjust the fit obtained by automated methods visually, using the sliders provided for each fit parameter (*Manual Fit* panel). The goodness-of-fit statistics are updated in real time and colour changes indicate whether the fit has improved or worsened. Also shown are the panels controlling which data set is to be worked on (*Dataset* panel) and which graphical properties are associated with a particular data set (*Display Properties* panel).

Graphical fitting of Gaussian functions enables the user to interactively position and fit a Gaussian function in the *Graph* panel (Figure 2). For all curve fitting applications, a manual option is also available which enables the user to adjust the fit by changing individual fit parameters with sliders. The goodness of fit statistics are updated in real time, with red and green colours indicating whether a change of parameter values has improved or worsened the fit (Figure 3).

For graphical visualisation of plots we have developed the *GraphPanel* class (available as source file with Javadoc documentation as Additional file 1). This class provides a versatile tool for plotting any two-dimensional data either as symbol or line graphs. All graphical features such as line cursors, zoom and integration windows, etc. are carried out by this class. For interpolation of functions in between discrete data points, a cubic spline algorithm has been implemented, following the numerical methods published in [6]. The *GraphPanel* class, since its inception, has been used in several of our other Java applications, including AFDP, ACDP, and DMAN [3,4].

Compared to other software applications in this area, the feature that distinguishes SDAR is its intuitive use and the emphasis on graphical operations. In contrast to the purely number-based handling of curve fitting and data transformations in other software, SDAR offers a graphical manipulation feature where possible, therefore enabling a visual interaction and assessment of the user with the plotted data.

An important aspect in the design of SDAR is to provide flexibility to the user by ensuring compatibility with other software. The program Grace has a long-standing history in data transformation and plotting, and we therefore decided to have data saved by SDAR in a Grace-compatible format. All data files saved within SDAR have a header section with Grace-compatible commands. There is also an option *Export to Grace* which will save all current data series in a Grace-compatible input file.

## Conclusions

SDAR is a simple-to-use, platform-independent visualisation and analysis tool for two-dimensional data implemented in Java. The program features and the graphical user interface have been designed considering convenient usage and flexible applications. Images can be generated directly from SDAR and saved as high quality SVG files, as well as in the highly portable PNG and TIFF formats. Data processed in SDAR can be exported in a format compatible with Grace.

For future versions of this software, we plan to implement further smoothing and curve fitting features as well as deconvolution. For improved output features, we plan to include PDF generation, as well as export to the gnuplot software ( http://www.gnuplot.info/).

## Availability and requirements

• **Project name:** SDAR

• **Project homepage**: http://www.structuralchemistry.org/pcsb/

• **Operating system**: OS-independent

• **Programming language**: Sun/Oracle Java version 1.6.0_03

• Other requirements: Sun/Oracle JRE 6 or JRE 7

• **Licence**: EULA

• Any restrictions to use by non-academic user: Software is free for non-commercial users

• SDAR v2.1 is included as Additional file 2

• The manual is included as Additional file 3

• The GraphPanel class is included as source with documentation as Additional file 1

## Abbreviations

PCSB: Program Collection for Structural Biology and Biophysical Chemistry; PDF: Portable Document Format; PNG: Portable Network Graphics; SDAR: Serial Data Arithmetic; SVG: Scalable Vector Graphics; TIFF: Tagged Image File Format.

## Competing interest

The authors declare that they have no competing interests.

## Authors’ contributions

AH invented, designed and wrote algorithms. SW and NJH contributed to the design and writing of algorithms. SW, NJH, AH and AS tested the program; SW and AS wrote the manual. All authors contributed to drafting the manuscript. All authors read and approved the final manuscript.

## Supplementary Material

Additional file 1 **Source file of the *GraphPanel *class with Javadoc documentation.** Click here for file

Additional file 2 The program compiled with Java 1.6.0. Click here for file

Additional file 3 The manual for SDAR. Click here for file
