# Supplementary material for: SDAR: a practical tool for graphical analysis of two-dimensional data
Source: BMC Bioinformatics. 2012 Aug 14;13:201. doi: 10.1186/1471-2105-13-201 (PMC3480940; doi:10.1186/1471-2105-13-201)
Supplement: Additional file 3 — The manual for SDAR. [file 1471-2105-13-201-S3.pdf]

SDAR v2.1

Table of Contents

1. SUMMARY 2

2. GENERAL LAYOUT AND OPERATION 2

2.1 How to load 2
2.2 How to save 3

3. FILE 3

3.1 Import data 3
3.2 Load 3
3.3 Save 3
3.4 Export to Grace 3
3.5 Write image 3
3.6 Exit 4

4. EDIT 4

4.1 Copy 4

5. FORMAT 4

5.1 View size 4
5.2 Plot properties 4
5.3 Axes properties 4
5.4 Show legend 4

6. TRANSFORM X 4

6.1 Translate X 4
6.2 Scale X 4
6.3 Change data pitch 5

7. TRANSFORM Y 5

7.1 Translate Y 5
7.2 Scale Y 5
7.3 Baseline linear 5
7.4 Smoothing 5
7.5 Dataset addition 5

8. ANALYSIS 6

8.1 Maxima 6
8.2 Minima 6
8.3 Integration 6

9. CURVE FITTING 6

9.1 Linear 6
9.2 Sigmoid 6
9.3 Hill 6
9.4 Hill with background 6
9.5 Dose Response 7
9.6 Gaussian 7
9.7 Exponential 7
9.8 Manual (Gaussian) 7

 © 1999-2012 by Hofmann Laboratory,

10. HELP......7

10.1 Licence......7
10.2 About......7

11. TOOLBAR......7

11.1 Datasets......7
11.2 Autoscale......7
11.3 Zoom......8
11.4 X Cursor......8
11.5 Y Cursor......8

12. STATISTICAL PARAMETERS......8

13. NON-PCSB JAVA LIBRARIES USED IN THIS PROGRAM......8

14. REFERENCES......9

15. APPENDIX 1: COPYRIGHT, LICENCE AND DISCLAIMER......10

1. Summary

SDAR (Serial Data Analysis and Regression) is a Java application for graphical analysis, transformation and fitting of two-dimensional data. Numeric data in the form of multi-column ASCII files can be read into the program and graphed as x-y-plots. Data are output in a two-column format compatible with Grace (http://plasma-gate.weizmann.ac.il/Grace/), and sessions can be saved. Images of the Graph panel can be output in SVG, PNG or TIFF format.

2. General layout and operation

SDAR uses tabbed panels to enable viewing of datasets. The main panel tabbed ***Graph*** shows graphical x-y-plots of the current datasets. For each dataset, a new tabbed panel is added with the name of the set showing as label in the tab. These latter panels show the spreadsheet format of the dataset, comprising of the x-y-data in the first columns, as well as any data derived from analysis in SDAR in the following columns. At the bottom of these panels, two functions are provided: ***Close*** will delete this dataset from the current session, ***Save*** writes the current dataset to an ASCII file compatible with the format of the program Grace; data derived from analysis in SDAR will be saved as remarks (indicated by #) at the top of the file. In the table view, the user can change data entries in the first two columns. With ***Update*** the amended data get plotted in the ***Graph*** panel (note that in order to save the amended data, the ***Save*** option still needs to be executed).
A movable tool bar allows quick access to frequently used graphics functions.
The current session can be saved in an ASCII file in SDAR format using the ***File-Save***, and retrieved with the ***File-Load*** function.
On the Graph panel, vertical and horizontal line cursors can be activated and positioned using either by click with the `left mouse button`, or the arrow keys `UP`, `DOWN`, `LEFT` and `RIGHT`. The x- and y-values of the active dataset (see 11.1) at the current cursor position are displayed in the status bar. Transformations can be applied simultaneously to any selection of datasets.

2.1 How to load

Data can be loaded into SDAR by

- importing x-y-data using the ***File-Import data*** option which uses a smart parser to find numerical data organised into columns in an ASCII file

- loading an existing file in SDAR-format using the ***File-Load*** option
- by starting the program with a data file name as argument in the command line.

2.2 How to save
Data processed in SDAR can be saved in different ways:
- Data can be saved using the ***File-Save*** option as an ASCII file in SDAR-format (the x-y-data is fully contained in this file, no separate files with individual dataseries are generated). This option will save all current dataseries at once.
- Data can be written out as an ASCII file fully compatible with Grace using the ***File-Export to Grace*** option. This option will save all current dataseries at once.
- Individual datasseries can be saved as ASCII files by using the ***Save*** option on the individual spreadsheet panes. These files will have remarks (indicated by #) with information from SDAR at the top. The remark section is followed by instructions for Grace (indicated by @). The x-y-data is then listed in two-column format.

3. File
3.1 Import data
Data from ASCII data files with multiple columns can be imported with this function. The parser will analyse the ASCII file as to the presence of a continuous section of a certain number of columns. If there are more than two columns, the user can select which column shall be assigned to *x*, *y* or *sigma*. The data type can be chosen as ***Continuous*** (displayed as line plot in the ***Graph*** panel), or ***Discrete*** (displayed as unconnected symbols in the ***Graph*** panel).
If the data file to be loaded has consecutive multiple y-values for the same x-value, the y-values are averaged and only one x-y-data pair is passed on to SDAR.

3.2 Load
With this function, a previous session saved with SDAR can be loaded.

3.3 Save
The currently active session can be saved into an ASCII file.

3.4 Export to Grace
Data can be written out as an ASCII file fully compatible with Grace using the ***File-Export to Grace*** option. This option will save all current dataseries at once. Relevant instructions for the Grace program will be at the top of the file (indicated by @).

3.5 Write image
The ***Graph*** panel can be saved as an image in the formats SVG, PNG or TIF. In order to generate scalable vector graphics (SVG) images in SDAR, the Apache Batik SVG toolkit (http://xmlgraphics.apache.org/batik/index.html) has been implemented. Other image formats are then generated from the initial SVG image by transcoders within Batik.
Note: Due to an apparent bug in Batik, transcoding to JPG images does currently not work. For future versions, we also plan to include a PDF transcoder using the Apache FOP toolkit (http://xmlgraphics.apache.org/fop).

3.6 Exit

This exits the program; changes to datasets the user has not saved will be lost.

4. Edit

4.1 Copy

This function copies the current content of the ***Graph*** panel into the clipboard. Note: Copy-paste does not work under Linux with OpenOffice as of Feb 2009 (http://bugs.sun.com/bugdatabase/view_bug.do?bug_id=6607163).

5. Format

5.1 View size

Sets the size of the ***Graph*** panel; default: 1075x526.

5.2 Plot properties

This allows setting the title and subtitle of the plot, including font type, size and style.

5.3 Axes properties

The titles of the x- and y-axis can be entered, and the scales and tick intervals for both axes can be set. The position of the left and right y-axes as well as the lower and upper x-axes can be changed by pressing the `left mouse button` at the respective axis and dragging it to its new location.

5.4 Show legend

If legend labels for the current datasets have been entered by the user, the legend box will be shown or hidden on ***Graph*** panel. Legend labels can be entered using the Dataset button from the tool bar (see 11.1). The legend box can be re-positioned by pressing the `left mouse button` inside the legend box and dragging it to its new location.

6. Transform X

6.1 Translate X

With this function, a translation of the datasets activated for transformation (see ***Transform*** in 11.1) along the x-axis can be achieved. This is may e.g. be useful to re-define the point of x=0 in a dataset.

A pop-up window allows the user to enter which new value (***New***) a current x-value (***Old***) should adopt; the numbers can be entered into the text fields. Alternatively, the vertical x-cursor is active and can be positioned by the user to indicate the ***Old*** x-value that should adopt the value specified in ***New***.

The transformation applied to the datasets is: $x_{new} = x_{old} + (\mathbf{New} - \mathbf{Old})$

6.2 Scale X

This allows the user to multiply a constant scale factor into all current x values; the scale factor is entered into a text field in a pop-up window. This applies to all datasets currently activated for transformation.

The transformation applied to the dataset is: $x_{new} = x_{old} * scale$

6.3 Change data pitch

This allows the user to change the data pitch of the currently active datasets. x-y-pairs that have a smaller Δx than the new data pitch to their preceding x-y-pair will be deleted. This applies to all datasets currently activated for transformation.

7. Transform Y

7.1 Translate Y

With this function, a translation of the datasets activated for transformation along the y-axis can be achieved. A pop-up window allows the user to enter which new value (***New***) a current y-value (***Old***) should adopt; the numbers can be entered into the text fields. Alternatively, the horizontal y-cursor is active and can be positioned by the user to indicate the ***Old*** y-value that should adopt the value specified in ***New***.
The transformation applied to the datasets is: $y_{new} = y_{old} + (\textbf{New - Old})$

7.2 Scale Y

This allows the user to multiply a constant scale factor into all current y values; the scale factor is entered into a text field in a pop-up window. This applies to all datasets currently activated for transformation.
The transformation applied to the dataset is: $y_{new} = y_{old} * scale$

7.3 Baseline linear

This function subtracts a linear function from all datasets activated for transformation. This is useful for example where baseline or drift corrections need to be carried out. A line with two squares at both ends will appear on the ***Graph*** panel and can be modified by the user as to the slope and y-position. To change the slope, the user needs to move either square up or down using the `left mouse button`. To change the y-position of the entire line, a vertical movement outside the squares is required.
The transformation applied to the dataset is: $y_{new} = y_{old} – (m * x + t)$, where m and t are determined from the line modified graphically by the user.

7.4 Smoothing

This function will open a pop-up window where the user can specify the smoothing type (***Mean*** or ***Median***), as well as the degree of smoothing using a slider. The smoothing algorithms perform local averaging of an equally spaced function using the local mean or median within the window of neighbouring points determined by the slider position. This applies to all datasets currently activated for transformation.

7.5 Dataset addition

Here, graph arithmetic can be performed, i.e. pairwise addition or subtraction of datasets. Using selected entries from two drop down lists, the second dataset will be added to the first dataset with a multiplication factor added in the text field between. SDAR will produce the resulting dataset based on the x-values and data pitch of the first dataset within the shared x-region of both datasets. y-values for the second dataset are calculated by cubic spline interpolation.

PCSB – Programme Collection for Structural Biology and Biophysical Chemistry © 1999-2012 by Hofmann Laboratory,
Structural Chemistry Program, Eskitis Institute for Cell & Molecular Therapies, Griffith University

8. Analysis

8.1 Maxima

With this menu item, an automatic determination of maxima in the currently active dataset will be performed. The maxima found by SDAR are highlighted in the ***Graph*** panel by blue vertical lines, and listed in pop-up window next to tick boxes. The user can accept or deny individual maxima by ticking/unticking. Further maxima can also be added manually, by clicking on ***Add Peak***; this will activate the green vertical x cursor that can be placed the user at the appropriate position. Hitting ENTER will include the current x-value in the list of maxima.

This procedure is required prior to attempt ***Curve fitting*** with ***Gaussian*** functions.

8.2 Minima

Minima determination is performed as described under 8.1 for maxima.

8.3 Integration

Data can be integrated within a selected interval. A pop-up window will open where the user can enter the start (***From X***) and end (***To X***) of the interval. Alternatively, pressing and dragging the left mouse button, the area to be integrated can be highlighted graphically in the ***Graph*** panel. Hitting ENTER or clicking ***OK*** will perform the integration. The algorithm used by SDAR is based on numerical integration by Simpson's rule.

9. Curve fitting

For curve fitting, the user can choose between linear gression, non-linear Melder-Nead Simplex or Levenberg-Marquardt minimisation, depending on the type of equation. All curves can also be fitted manually using the sliders provided next to each parameter in the ***Manual Fit*** panel. If multiple datasets are loaded, the dataset to be fitted needs to be selected as ***Active*** under ***Datasets*** (11.1).

In the following cases, a pop-up window will open that allows the user to enter the fitting area (Fit from X, to X) and initial values for the fitting parameters. SDAR will provide an initial guess for all parameters. Individual parameters can be kept fixed during the minimisation calculation by unticking the ***Fit*** checkbox.

9.1 Linear

This will fit a linear equation: $y = m * x + t$, using either linear regression, Levenberg-Marquardt minimisation or manual fit.

9.2 Sigmoid

This will fit a linear equation: *y= Ampl / {1 + e^[- (x – Infl) / Width]} + Int*, using either non-linear Simplex, Levenberg-Marquardt minimisation or manual fit.

9.3 Hill

This will fit a Hill equation: $y = A * x^n / (k^n + x^n)$, using either non-linear Simplex, Levenberg-Marquardt minimisation or manual fit.

9.4 Hill with background

This will fit a Hill equation with optional linear background: $y = A * x^n / (k^n + x^n) + (m * x + t)$, using either non-linear Simplex, Levenberg-Marquardt minimisation or manual fit.

9.5 Dose Response
This will fit a logistic EC50 equation: $y= bottom + (top - bottom) / [1 + (x / EC50)^{-slope}]$, using either non-linear Simplex or manual fit.

9.6 Gaussian
Fitting of one or multiple Gaussian functions requires the prior definition of maxima (8.1). Then, the sum of Gaussians can be fitted using the equation: $y= \Sigma \{Ampl * e\text{^}[- (x- Centre) / Width]\}$. The user can choose between either non-linear Simplex, Levenberg-Marquardt minimisation or manual fit.

9.7 Exponential
This will fit a linear equation: $y= a * e\text{^}(-\lambda * x) + b$, using either non-linear Simplex, Levenberg-Marquardt minimisation or manual fit.

9.8 Manual (Gaussian)
Gaussian functions can be fitted to data graphically by performing a right-click with the mouse where the centre of the Gaussian should be. The centre can then be moved by pressing the left mouse button on the maximum of the displayed curve and dragging it to desired position; vertical dragging will increase the amplitude. Pressing the left button of the mouse while pointing to either one of the two half-maxima of the curve will widen the Gaussian. A pop-up window will inform about the current parameters of the fitted Gaussians and the fit statistics to the data.

10. Help
10.1 Licence
Shows the licence conditions.

10.2 About
Displays the program version.

11. Toolbar
11.1 Datasets
This button will open a pop-up window with a list of currently loaded datasets. One of the datasets can be selected as ***Active*** and enable evaluation of cursor values displayed in the status bar underneath the ***Graph*** panel. This function is also need to select the dataset to be fitted.
Multiple datasets can be selected for transformation operations by ticking the ***Transformation*** box next to each individual dataset. Under ***Properties***, buttons with the current graph colour are being displayed. Clicking on these buttons will open another window that allow the user to change the colour, line or symbol settings, and enter a description for the legend.

11.2 Autoscale
This button will auto-scale the plots in the Graph panel to display all data with respect to x and y.

11.3 Zoom
After clicking this button, the user can drag open (left mouse button) a rectangle on the ***Graph*** panel which will re-define the boundaries of the plot area to be shown.

11.4 X Cursor
This button activates the vertical line cursor that can be positioned with a click on the `left mouse button` or the `LEFT ARROW / RIGHT ARROW` keys. In the status bar, the current x- and y-values of the ***Active*** dataset will be displayed. A second click on this button will de-activate the x-cursor.

11.5 Y Cursor
This button activates the horizontal line cursor that can be positioned with a click on the `left mouse button` or the `UP ARROW / DOWN ARROW` keys. Where the y-cursor intersects with the currently active dataset, vertical drop lines are displayed. In the status bar, the current y-value will be displayed. A second click on this button will de-activate the y-cursor.

12. Statistical parameters
Fit statistics are calculated to assess the goodness of fit between the fitted function and the loaded ("experimental") data. The following parameters are calculated within the boundaries of the fitted region (Fit from X, to X):

R-factor= $\Sigma\ |y_{exp}| - |y_{fit}|\ /\ \Sigma\ |y_{exp}|$
A perfect fit has an R-factor of 0.

$R^2 = \Sigma\ (y_{fit} - \mu_{exp})^2\ /\ \Sigma\ (y_{exp} - \mu_{exp})^2$, with the average $\mu_{exp} = 1/n * \Sigma\ y_{exp}$
A perfect fit has an R^2 parameter of 1.

SSE= $\Sigma\ (y_{exp} - y_{fit})^2$
Summed square error (unweighted χ^2). A perfect fit has an SSE of 0.

$\chi^2 = \Sigma\ [(y_{exp} - y_{fit})\ /\ \sigma_{exp}]^2$
If the loaded data does not include weights, χ^2 is automatically computed as unweighted χ^2 (and therefore identical to SSE). A perfect fit has a χ^2 of 0.

13. Non-PCSB Java libraries used in this program
SDAR makes use of the following Java libraries not authored by us:

Apache Batik SVG toolkit
http://xmlgraphics.apache.org/batik/index.html

Apache FOP toolkit
http://xmlgraphics.apache.org/fop/

Nelder-Mead Simplex and linear regression algorithms have been extracted from the Regression libraries by M. Flanagan
http://www.ee.ucl.ac.uk/~mflanaga/java/

Levenberg-Marquardt in Java by J. P. Lewis
http://scribblethink.org/index.html

14. References

When using SDAR, please cite (Hofmann & Wlodawer, 2002).

Hofmann A. & Wlodawer A. (2002) PCSB - a program collection for structural biology and biophysical chemistry. *Bioinformatics* **18**, 209-210.

15. Appendix 1: Copyright, Licence and Disclaimer

COPYRIGHT
Copyright (c) 1999-2012 by
Hofmann Laboratory, Structural Chemistry Program
Eskitis Institute for Cell & Molecular Therapies, Griffith University.
http://www.structuralchemistry.org/pcsb/
All rights reserved.

LICENCE

The authors grant you a non-exclusive, royalty-free licence to use this software, provided that

i) the software is only used for not-for-profit applications;
ii) you do not decompile, reverse engineer or modify the object code;
iii) you do not utilise the software in a manner which is disparaging to the authors;
iv) usage of this software is properly cited.

DISCLAIMER

This software is provided "as is", without a warranty of any kind. All express or implied conditions, representations and warranties, including any implied warranty of merchantability, fitness for a particular purpose or non-infringement, are hereby excluded. The authors shall not be liable for any damages suffered by the user as a result of using or distributing the software or its derivatives. In no event will the authors be liable for any lost revenue, profit or data, or for direct, indirect, special, consequential, incidental or punitive damages, however caused and regardless of the theory of liability, arising out of the use of or inability to use software, even if the authors have been advised of the possibility of such damages.
